# Loss of ZNF677 Expression Is an Independent Predictor for Distant Metastasis in Middle Eastern Papillary Thyroid Carcinoma Patients

**DOI:** 10.3390/ijms22157833

**Published:** 2021-07-22

**Authors:** Abdul K. Siraj, Pratheesh Kumar Poyil, Sandeep Kumar Parvathareddy, Khadija Alobaisi, Saeeda O. Ahmed, Saif S. Al-Sobhi, Fouad Al-Dayel, Khawla S. Al-Kuraya

**Affiliations:** 1 Human Cancer Genomic Research, Research Center, King Faisal Specialist Hospital and Research Center, P.O. Box 3354, Riyadh 11211, Saudi Arabia; asiraj@kfshrc.edu.sa (A.K.S.); ppoyil@kfshrc.edu.sa (P.K.P.); psandeepkumar@kfshrc.edu.sa (S.K.P.); kalobaisi@kfshrc.edu.sa (K.A.); ahmsaeeda@kfshrc.edu.sa (S.O.A.); 2 Department of Surgery, King Faisal Specialist Hospital and Research Center, P.O. Box 3354, Riyadh 11211, Saudi Arabia; sobhi@kfshrc.edu.sa; 3 Department of Pathology, King Faisal Specialist Hospital and Research Center, P.O. Box 3354, Riyadh 11211, Saudi Arabia; dayelf@kfshrc.edu.sa

**Keywords:** zinc finger protein 677, papillary thyroid carcinoma, distant metastasis, tumor suppressor

## Abstract

Thyroid cancer incidence has increased in recent decades. Papillary thyroid carcinoma (PTC) is the most common type of thyroid cancer. Approximately 30% of PTC patients develop recurrence or distant metastasis and tend to have poor prognosis. Therefore, the identification of targetable biomarkers in this subset of patients is of great importance. Accumulating evidence indicates that zinc finger protein 677 (ZNF677), which belongs to the zinc finger protein family, is an important effector during the progression of multiple malignancies. However, its role in Middle Eastern PTC patients has not been fully illustrated. Here, we uncovered the molecular mechanism and the clinical impact of ZNF677 expression in a large cohort of more than 1200 Middle Eastern PTC and 15 metastatic tissues. We demonstrated that ZNF677 is frequently downregulated in primary PTC (13.6%, 168/1235) and showed that complete loss of expression of ZNF677 is significantly associated with aggressive clinico-pathological markers such as extrathyroidal extension (*p* = 0.0008) and distant metastases (*p* < 0.0001). We also found a significantly higher incidence of ZNF677 loss in primary tumors with distant metastases (33.3%; *p* < 0.0001) as well as in distant metastatic tissues (46.7%; *p* = 0.0002) compared to the overall cohort (13.6%). More importantly, PTC with loss of ZNF677 expression showed significantly lower metastasis-free survival (*p* = 0.0090). Interestingly, on multivariate logistic regression analysis, ZNF677 loss was an independent predictor of distant metastasis in PTC (Odds ratio = 2.60, 95% Confidence interval = 1.20–5.62, *p* = 0.0155). In addition, we found a significant association between ZNF677 loss and phospho-AKT expression (*p* < 0.0001). Our functional molecular results suggest that ZNF677 acts as a tumor suppressor, mediating its effect by inhibiting AKT phosphorylation. Taken together, our results highlight the pivotal role played by ZNF677 during carcinogenesis and metastasis formation in Middle Eastern PTC patients.

## 1. Introduction

Thyroid cancer is the most common endocrine malignancy, with a recent rapid increase in its incidence [1,2,3,4]. Papillary thyroid carcinoma (PTC) accounts for the majority of thyroid cancers and is known to have favorable prognosis [3,5,6]. However, up to 15% of PTC patients were found to develop distant metastases [7,8,9,10] after initial therapy, which was the main cause of thyroid cancer-related deaths [11,12,13,14]. Disease-specific mortality rates at 10 years for patients with distant metastasis is 70% [15]. Therefore, distant metastasis is considered the most important prognostic factor for PTC patients. The American Thyroid Association guidelines identify PTC patients with distant metastasis as high-risk patients who require more aggressive treatments [16]. Therefore, exploring the molecular markers that can predict metastasis in PTC patients is critical for diagnosis and early therapeutic interventions.

Zinc finger proteins (ZNF) are large transcription factors with a “finger-like” domain that are known to be involved in important cellular biological process [17,18]. The majority of ZNFs belong to the Krupple associated box domain zinc finger protein (KRAB-ZNF) superfamily, which is known to contribute and regulate cellular growth, differentiation, migration, and invasion [19,20]. Most of the KRAB-ZNFs are involved in epigenetic silencing that could be mediated by DNA methylation [21,22]. An elevated methylation level of tumor suppressor genes is considered an important hallmark of cancer [23,24,25]. ZNF677, a member of the KRAB-ZNF super family, is encoded in the KRAB-ZNF cluster on chromosome 19q [26,27]. It was reported to be frequently methylated in human cancers [28,29]. Moreover, hypermethylation of *ZNF677* was reported to decrease ZNF677 expression, which could serve as a prognostic markers in several malignancies including lung cancer [28] and thyroid cancer [29]. Previous evidence revealed that ZNF677 exerts its suppressor functions in tumor cells through interfering with phosphorylation and activation of AKT [29].

The PI3K/AKT pathway can regulate the function of a number of cellular proteins involved in cellular proliferation, apoptosis, and metastasis [30]. AKT is a key component of the PI3K pathway, and its activation by genetic or epigenetic mechanisms will provide cancer cells with growth advantage and metastatic competence. Although previous attempts to identify the prognostic impact of ZNF expression in PTC using mRNA expression revealed its negative prognostic impact [29], little is known about the association of ZNF677 expression with clinico-pathological characteristics and its prognostic significance for PTC in relation to ethnicity.

Thus, we conducted this study to analyze the protein expression of ZNF677 in a large cohort of more than 1200 PTC and 200 normal thyroid tissues, as well as the association of ZNF677 expression with clinico-pathological markers and patient survival. We found not only that ZNF677 was more highly expressed in normal thyroid tissue than in tumor tissue, but also that loss of its expression was predictive of distant metastasis. Additionally, we investigated ZNF677 expression and methylation in PTC cell lines. We analyzed tumor cell growth-suppressing properties of ZNF677 and its effects on proliferation and migration of PTC cells. Besides, mechanistic studies have pinpointed AKT phosphorylation as a major signaling mechanism functioning downstream of ZNF677-regulated cell growth. Overall, our results indicate that ZNF677 is a potential tumor suppressor, and its loss of expression may lead to the activation of tumor-promoting pathways in PTC. Moreover, ZNF677 expression is frequently altered in Middle Eastern PTC, with detrimental effects on patient survival and outcome.

## 2. Results

### 2.1. ZNF677 Expression and Its Clinico-Pathological Associations

**Figure 1:**
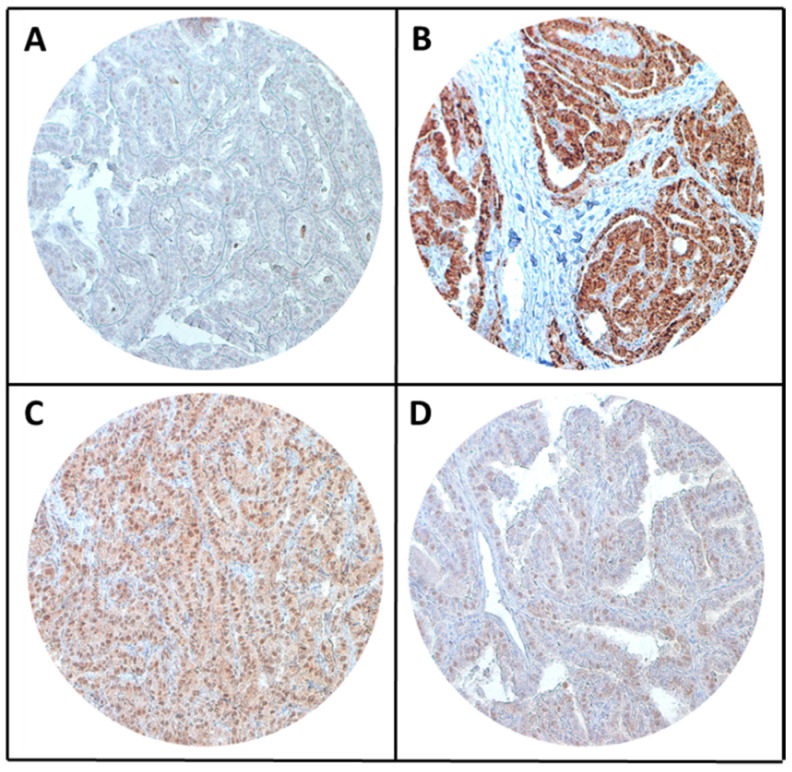
Tissue microarray (TMA)-based immunohistochemistry analysis of ZNF677 and phospho-AKT in papillary thyroid carcinoma (PTC) patients. PTC TMA spots showing loss of ZNF677 (**A**) and low expression of phospho-AKT (**D**). In contrast, another set of TMA spots shows normal expression of ZNF677 (**B**) and high expression of phospho-AKT (**C**). 20 X/0.70 objective on an Olympus BX 51 microscope. (Olympus America Inc., Center Valley, PA, USA).

**Figure 2:**
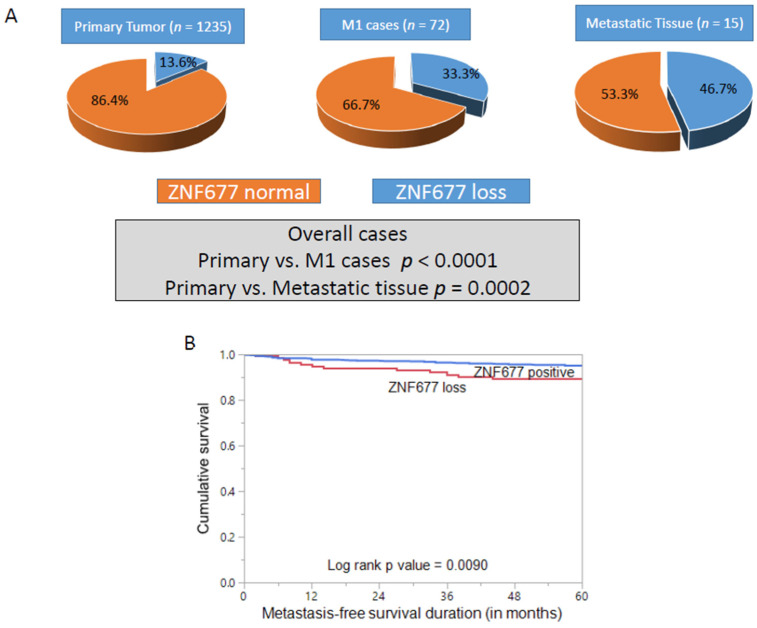
(**A**) Increasing proportion of ZNF677 loss in different PTC cohorts. ZNF677 loss was noted in 13.6% of primary tumors, 33.3% of M1 cases, and 46.7% of metastatic tissues. The difference in the frequency of ZNF677 loss between primary tumor and M1 cases as well as primary tumor and metastatic tissues was statistically significant (*p* < 0.0001 and *p* = 0.0002, respectively). (**B**) Metastasis-free survival (MFS) analysis of ZNF677 protein expression in PTC. Kaplan–Meier survival plot showing statistically significant poor MFS in cases with ZNF677 loss compared to ZNF677-positive cases (*p* = 0.0090).

The clinico-pathological characteristics of the study population are presented in Table 1. Loss of ZNF677 protein expression was noted in 13.6% (168/1235) of PTCs in our cohort (Figure 1). We also analyzed 223 normal thyroid tissues and found normal expression of ZNF677 in all of them. ZNF677 loss in PTC was significantly associated with the follicular variant of PTC (*p* < 0.0001), extrathyroidal extension (*p* = 0.0008), and distant metastasis (*p* < 0.0001) (Table 2). We also found a significant inverse association between ZNF677 loss and phospho-AKT expression (*p* < 0.0001). Since we found a significant association of ZNF677 loss with distant metastasis, we further analyzed the incidence of ZNF677 loss in primary cases with distant metastasis (*n* = 72) and found a statistically significant difference in the incidence of ZNF677 loss between this subset of patients and the overall cohort (33.3% vs. 13.6%, *p* < 0.0001). Furthermore, ZNF677 expression was also determined in 15 PTC cases for which distant metastatic tissue samples were available. ZNF677 loss was noted in 46.7% (7/15) of metastatic tissues, and the difference in the incidence of ZNF677 loss with respect to the overall cohort was statistically significant (46.7% vs. 13.6%, *p* = 0.0002) (Figure 2A).

We next sought to determine the prognostic significance of ZNF677 in our cohort. Cases showing loss of ZNF677 had a worse metastasis-free survival (MFS_ compared to cases with normal expression (*p* = 0.0090) (Figure 2B). More importantly, on multivariate logistic regression analysis, ZNF677 loss was an independent predictor of distant metastasis in PTC (odds ratio = 2.60, 95% CI = 1.20–5.62, *p* = 0.0155) (Table 3).

### 2.2. ZNF677 Impedes PTC Cell Growth In Vitro

**Figure 3:**
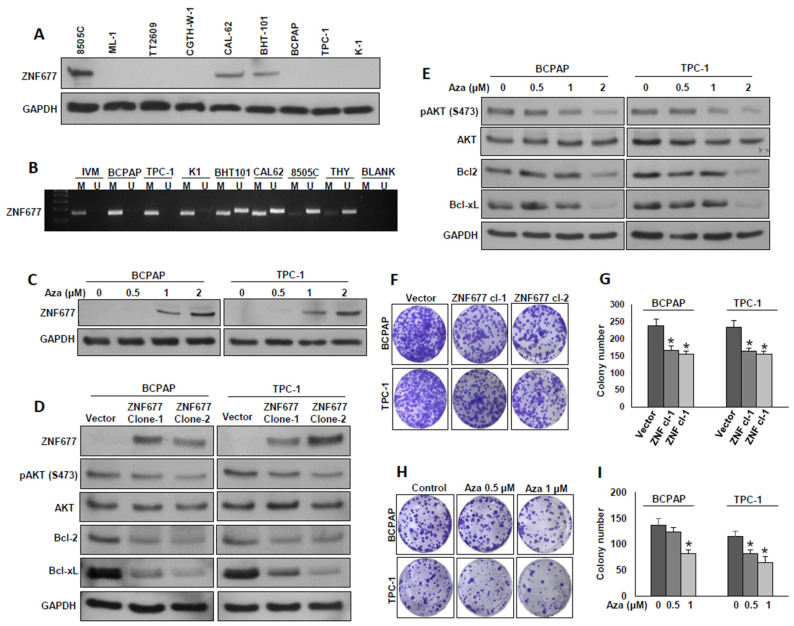
ZNF677 inhibits PTC cell growth in vitro. (**A**) Basal expression of ZNF677 in a panel of thyroid cancer cell lines. Proteins were isolated from nine thyroid cancer cell lines and immunoblotted with antibodies against ZNF677 and GAPDH *(n* = 3). (**B**) Methylation status of thyroid cancer cell lines assessed by methylation-specific PCR for the *ZNF677* gene. MSP analyses of both methylated (M) and unmethylated (U) reactions were amplified from bisulfite-treated DNA and run in a 2% agarose gel. Distinguishable unmethylated (8505C), methylated (BCPAP, TPC-1, and K1), and partially methylated (BHT-101 and CAL62) bands can be seen in the gel. (**C**) Demethylation of the *ZNF677* gene restored ZNF677 expression in BCPAP and TPC-1 cells. PTC cells were treated with different doses (0.5, 1, and 2 µM) of 5-aza-2′deoxycytidine for 72 h before lysis. Equal amounts of proteins were immunoblotted with antibodies against ZNF677 and GAPDH (*n* = 3). (**D**) Ectopic expression of ZNF677 downregulates AKT phosphorylation and anti-apoptotic protein expression. BCPAP and TPC-1 cells were transfected with either empty vector or with ZNF677 cDNA, and overexpressing clones were selected and immuno-blotted with antibodies against ZNF677, pAKT, AKT, Bcl-2, Bcl-xL, and GAPDH as indicated (*n* = 3). (**E**) Demethylation of *ZNF677* gene downregulates AKT phosphorylation and anti-apoptotic protein expression. PTC cells were treated with the indicated doses of 5-aza-2′deoxycytidine for 72 h before lysis. Equal amounts of proteins were immunoblotted with antibodies against pAKT, AKT, Bcl2, Bcl-xL, and GAPDH (*n* = 3). (**F**,**G**) Forced expression of ZNF677 decreases clonogenicity. ZNF67- overexpressing clones were seeded at a density of 500 cells per well in a 6-well plate and grown for an additional 10 days, then stained with crystal violet, and colonies were counted. (**H**,**I**) Demethylation of ZNF677 gene decreases clonogenicity. PTC cells (500 cells per well in a 6-well plate) were treated with the indicated doses of 5-aza-2′deoxycytidine for 72 h and grown for an additional 10 days, then stained with crystal violet, and colonies were counted. Data presented in the bar graphs are the mean ± SD of three independent experiments (*n* = 3). * Indicates a statistically significant difference compared to control with *p* < 0.05.

In an attempt to explore the role of ZNF677 in PTC cell growth, we first analyzed the basal expression of ZNF677 in a panel of nine thyroid cancer cell lines by immunoblotting (Figure 3A and Supplementary Figure S1A). Based on ZNF677 expression, we identified three ZNF677-expressing thyroid cancer cell lines (8505C, Cal-62, and BHT-101) and six thyroid cancer cells with loss of ZNF677 expression (ML-1, TT609, CGHT-W1, BCPAP, TPC-1, and K1). Interestingly, we found complete loss of ZNF677 expression in all the three PTC cell lines tested (Figure 3A).

Previous studies report that ZNF677 is frequently silenced by promoter methylation in non-small cell lung cancer [28] and thyroid cancer [29]. To test this, the methylation status of ZNF677 promoter region in PTC cell lines were analyzed using Methylation-specific PCR (MSP). As shown in Figure 3B, there was a complete methylation of the ZNF677 gene promoter in three PTC cell lines (BCPAP, TPC-1, and K1). In an effort to restore methylated ZNF677, BCPAP and TPC-1 cells were treated with different doses (0.5, 1 and 2 µM) of 5-aza-2′-deoxycytidine, a demethylating agent, for 72 h. Demethylation restored ZNF677 protein expression in BCPAP and TPC-1 cells, as detected by immunoblotting (Figure 3C and Supplementary Figure S1B). These data suggest that loss of ZNF677 protein expression is a direct consequence of ZNF677 methylation.

Our clinical data showed that loss of ZNF677 expression is significantly associated with AKT phosphorylation (S473). In addition, a previous report showed that ZNF677 inhibits AKT phosphorylation in thyroid cancer [29]. To study this inverse association in vitro, we overexpressed ZNF677 in BCPAP and TPC-1 cells and evaluated the expression of ZNF677, phospho-AKT (S473), and total AKT by immuno-blotting. As shown in Figure 3D, ectopic expression of ZNF677 markedly downregulated AKT phosphorylation in both cell lines without altering total AKT expression (Supplementary Figure S1C). Similarly, demethylation using 5-aza-2′-deoxycytidine restored ZNF677 protein expression, with concomitant downregulation of AKT phosphorylation in both PTC cell lines in a dose-dependent course (Figure 3E and Supplementary Figure S1D). In addition, forced expression of ZNF677 or demethylation of ZNF677 gene decreased the expression of the anti-apoptotic proteins Bcl2 and Bcl-xL (Figure 3D,E). Next, we determined the effect of ectopic expression of ZNF677 or demethylation on PTC cell growth by the clonogenic assay. Forced expression of ZNF677 (Figure 3F,G) or demethylation of the *ZNF677* gene using 5-aza-2′-deoxycytidine (Figure 3H,I) significantly decreased cell growth. These data demonstrate that ZNF677 impedes PTC cell growth in vitro.

### 2.3. Ectopic Expression of ZNF677 Potentiates TRAIL-Induced Apoptosis in PTC Cells

**Figure 4:**
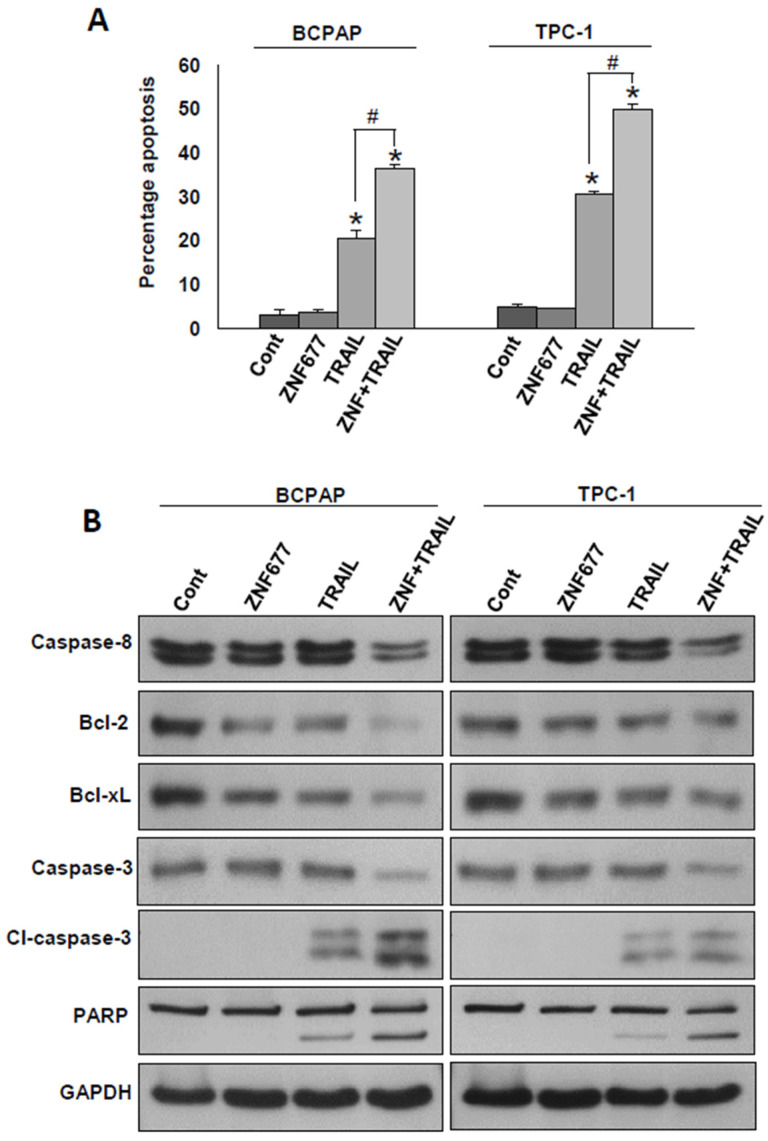
Forced expression of ZNF677 potentiates TRAIL-induced apoptosis in PTC cells. BCPAP and TPC-1 cells were stably transfected with either empty vector or ZNF677 cDNA. Stably transfected cell lines were treated with TRAIL (10 ng/mL) for 48 h. (**A**) Cells were stained with fluorescein-conjugated annexin-V and propidium iodide (PI) and analyzed by flow cytometry. Data presented by the bar graphs are the mean ± SD of three independent experiments (*n* = 3). * indicates statistically significant compared to empty vector control, # indicates statistically significant compared to TRAIL alone, with *p* < 0.05. (**B**) Cells were lysed, and equal amounts of proteins were immunoblotted with antibodies against caspase-8, Bcl-2, Bcl-xL, caspase-3, cleaved caspase-3, PARP, and GAPDH.

Our clinical and in vitro data suggest that ZNF677 is likely a tumor suppressor. Therefore, we wanted to determine whether ectopic expression of ZNF677 could potentiate TRAIL-induced apoptosis in PTC cells. For this, PTC cells after ZNF677 overexpression were treated with TRAIL (10 ng/mL) for 48 h and analyzed for apoptosis. As shown in Figure 4A, ZNF677 overexpression alone was not able to induce apoptosis in BCPAP and TPC-1 cell lines; however, significant apoptosis was observed after treatment with TRAIL alone of BCPAP (20.5 ± 1.8%) and TPC-1 (30.6 ± 0.67%) cell lines. Interestingly, ZNF677 overexpression followed by TRAIL treatment significantly increased the apoptotic population in BCPAP (36.6 ± 0.9%) and TPC-1 (50.09 ± 0.99%) cell lines. To confirm the above findings, we performed immunoblotting after treatment with and without TRAIL for 48 h of ZNF677- (Myc-DDK tagged) or empty vector-transfected PTC cell lines. As shown in Figure 4B, ZNF677 overexpression followed by TRAIL treatment markedly increased the cleavage of caspases-8 and -3 and PARP, as well as downregulated Bcl-2 and Bcl-xL expression in BCPAP and TPC-1 cells (Supplementary Figure S2).

### 2.4. ZNF677 Decreases the Metastatic Potential of PTC Cells

**Figure 5:**
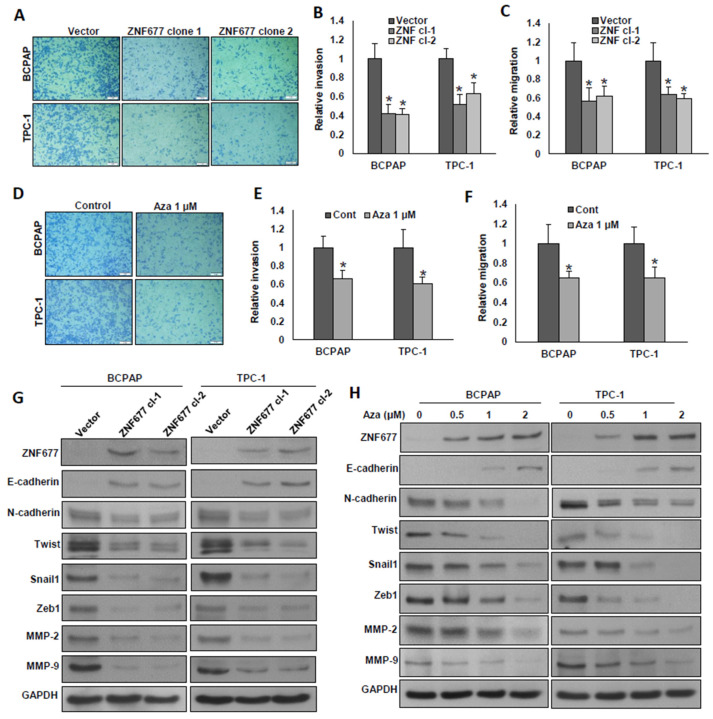
ZNF677 attenuates the metastatic potential of PTC cells. (**A**–**C**) Forced expression of ZNF677 decreased cell invasion and migration. ZNF677-overexpressing clones were seeded into the upper compartment of invasion or migration chambers. The bottom chambers were filled with RPMI media. After 24 h of incubation, invaded or migrated cells were fixed, stained, and quantified. (**D**–**F**) Demethylation of the *ZNF677* gene decreased cell invasion and migration. PTC cells after treatment with the indicated dose of 5-aza-2′deoxycytidine for 72 h were used for invasion and migration experiments as described before. Data presented by the bar graphs are the mean ± SD of three independent experiments (*n* = 3). * Indicates a statistically significant difference compared to control, with *p* < 0.05. (**G**,**H**) Forced expression of ZNF677 or demethylation of *ZNF677* gene attenuates EMT in PTC cells. Protein extracts from ZNF677-overexpressing clones or cells after treatment with the indicated dose of 5-aza-2′deoxycytidine for 72 h were immuno-blotted with antibodies against ZNF677, E-cadherin, N-cadherin, Twist, Snail1, Zeb1, MMP-2, MMP-9, and GAPDH (*n* = 3).

We demonstrated in our PTC patient cohort that complete loss of ZNF677 protein expression is significantly associated with aggressive clinico-pathological parameters such as extrathyroidal extension and distant metastasis. Therefore, we investigated whether ectopic expression of ZNF677 or demethylation of the *ZNF677* gene using 5-aza-2′-deoxycytidine could inhibit invasion, migration, and progression of epithelial-to-mesenchymal transition (EMT) in PTC cells. Forced expression of ZNF677 significantly decreased invasion (Figure 5A,B) and migration (Figure 5C) of PTC cells. As expected, demethylation also reduced the invasive (Figure 5D,E) and migratory (Figure 5F) potential of these cells. Ectopic expression of ZNF677 markedly down-regulated the expression of N-cadherin, Twist, Snail1, Zeb1, MMP-2, and MMP-9, with an accompanying expression of E-cadherin in both PTC cell lines (Figure 5G and Supplementary Figure S3A). Similar results were observed in PTC cells after demethylation using 5-aza-2′-deoxycytidine (Figure 5H and Supplementary Figure S3B). These data demonstrate that ZNF677 attenuates the metastatic potential of PTC cells.

### 2.5. Inhibition of AKT Decreases Cell Growth and Attenuates EMT in PTC Cells

**Figure 6:**
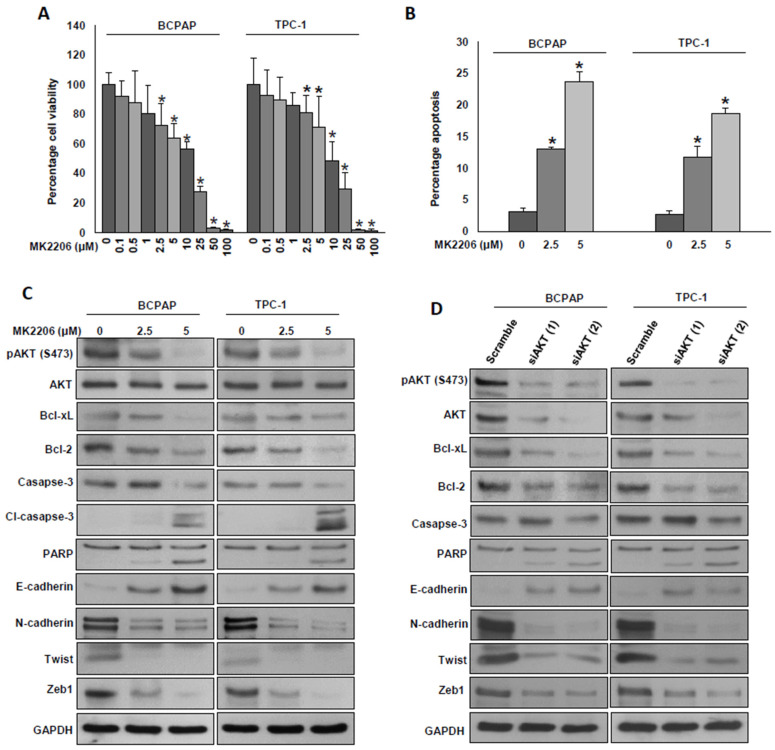
Inhibition of AKT decreases cell growth and attenuates EMT in PTC cells. (**A**) MK2206 induced cell viability loss in PTC cells. PTC cells were incubated with different doses of MK2206 for 48 h, and cell viability was determined by the MTT assay (*n* = 6), * *p* < 0.05. (**B**) MK2206-mediated apoptosis in PTC cells. PTC cells were treated with the indicated doses of MK2206 for 48 h, were subsequently stained with fluorescein-conjugated annexin-V and propidium iodide, and analyzed by flow cytometry. Data presented by the bar graphs are the mean ± SD of three independent experiments (*n* = 3). * Indicates a statistically significant difference compared with control, with *p* < 0.05. (**C**,**D**) Inhibition of AKT reduced the markers of cell growth and EMT in PTC cells. PTC cells were treated with the indicated doses of MK2206 or transfected two different AKT siRNAs (100 nM) for 48 h. Cells were lysed, and equal amounts of proteins were immunoblotted with antibodies against pAKT, AKT, Bcl-2, Bcl-xL, caspase-3, cleaved caspase-3, PARP, E-cadherin, N-cadherin, Twist, Zeb1, and GAPDH (*n* = 3).

We showed an inverse correlation between ZNF677 protein expression and AKT phosphorylation in both PTC patient tissue samples and PTC cell lines. Next, we sought to determine whether the inhibition of cell growth and metastasis by ZNF677 is mediated through the AKT pathway. For this, we inhibited AKT using siRNAs and a specific inhibitor, MK2206, and investigated cell growth and marker proteins of apoptosis and EMT. We treated PTC cell lines with increasing doses of MK2206 for 48 h and analyzed cell viability by the MTT assay. As shown in Figure 6A, there was a significant (*p* < 0.05) decrease in cell viability in both PTC cell lines in a dose-dependent manner. To determine whether loss of cell viability was due to apoptosis, PTC cells were treated with MK2206 (2.5 and 5 µM) for 48 h, stained with annexin V, and analyzed by flow cytometry. As shown in Figure 6B, MK2206 treatment significantly induced apoptosis in both PTC cell lines. Next, we tested the effect of AKT inhibition on marker proteins of apoptosis and EMT progression. As shown in Figure 6C,D, inhibition of AKT using MK2206 or siRNAs markedly downregulated the phosphorylation of AKT, anti-apoptotic proteins, Bcl2, and Bcl-xl and induced the cleavage of caspase-3 and PARP (Supplementary Figure S4A,B). AKT inhibition also decreased the expressions of N-cadherin, Twist, and Zeb1, with an accompanying expression of E-cadherin in both PTC cell lines (Figure 6C,D). These data show that the effect of ZNF677 on cell growth and metastasis is AKT-dependent.

## 3. Discussion

Distant metastasis is one of the most prominent features of malignancies and has great influence on patient survival. Distant metastasis is a very complex process that can be affected by genetic and epigenetic modifications and accounts for more than 90% of cancer-related death [31,32]. Several clinico-pathological factors have been identified as risk factors for distant metastasis [16,33,34]. In comparison, few studies have investigated the role of tumor suppressors and epigenetics in distant metastasis formation [35,36,37,38,39]. Our research indicated that ZNF677 expression can independently predict distant metastasis in Middle Eastern PTC patients.

Studies have shown that zinc finger ZNF677 is regulated by promoter methylation and plays an important role in human cancer [28,29]. A study by Li et al. [29] has demonstrated that ZNF677 is a tumor suppressor in thyroid cancer, and its down-regulation was associated with poor prognosis in a relatively small cohort of PTC. However, detailed clinico-pathological evaluation of ZNF677 expression in a large cohort of PTC has not been fully explored.

In this study, we demonstrated the clinical significance of ZNF677 expression in more than 1200 Middle Eastern PTCs. Increased frequency of ZNF677 loss of expression was noted in PTC tissues when compared with normal thyroid tissues. Most importantly, loss of ZNF677 expression correlated with adverse clinico-pathological factors including distant metastasis and presence of extrathyroidal extension. In addition, ZNF677 protein loss was associated with poor metastasis-free survival. Interestingly, this association remained significant even on multivariate analysis where other clinico-pathological prognostic markers were included.

The observation that loss of ZNF677 expression is mostly observed in the context of aggressive PTC and results in worsening the clinical outcome raised the intriguing possibility that ZNF677 alteration might not play a significant role in the initial tumorigenesis of PTC but rather contribute to its malignant progression and the formation of distant metastases. To test this hypothesis, we selectively analyzed an independent cohort of 72 PTC patients with documented distant metastasis. Our study confirmed that the frequency of ZNF677 expression loss in this independent cohort was 2.4 times higher than in the original cohort. Further confirmation of ZNF677 role in Middle Eastern PTC was found upon testing an additional cohort of 15 PTC patients with available metastatic tissue that revealed that ZNF677 expression loss was significantly much higher than in the overall cohort (46.7% vs. 13.6%, *p* = 0.0002). Interestingly, we also found that ZNF677 loss was an independent predictor of distant metastasis and could serve as a molecular biomarker to predict distant metastasis in Middle Eastern PTC. Our results indicate that more aggressive PTC tended to have higher frequency of ZNF677 loss, which further reflects the important role of this protein in advanced metastatic PTC.

Functionally, we confirmed that ZNF677 was downregulated and inactivated by promoter methylation using MSP assays and that demethylation restored ZNF677 expression in papillary thyroid cancer cells, which is in accordance with previous reports [28,29]. A previous study demonstrated that ZNF677 inhibits proliferation, migration, and invasion of thyroid cancer cells and exerts its tumor suppressor functions by inhibiting the phosphorylation of AKT via transcriptional repression of its two downstream targets, CDKN3 and HSPB1 (or HSP27) [29]. Mechanistically, we demonstrated that ectopic expression of ZNF677 markedly inhibited proliferation, migration, invasion, and EMT progression of papillary thyroid cancer cells. We also showed that ZNF677 exerted its effect via modulation of EMT and activation of the AKT cascade. These in vitro data provide additional evidence supporting a tumor suppressor role for ZNF677 in PTC.

Despite the strength of our study consisting in conducting ZNF677 analysis in a large cohort of Middle Eastern PTC and in the inclusion of an additional validation cohort, we acknowledge few limitations, which are the absence of in vivo studies and the lack of methylation data for PTC samples, since our methylation analysis was done only on PTC cell lines. Future studies are needed to verify that methylation is the main cause of ZNF677 inactivation in PTC patients of this ethnicity.

## 4. Materials and Methods

### 4.1. Sample Selection

One thousand two-hundred and thirty-five PTC patients diagnosed between 1989 and 2015 at King Faisal Specialist Hospital and Research Center (Riyadh, Saudi Arabia) with available archival tissue samples were included in the study. We also included 15 PTC cases for which distant metastatic tissues were available (10 from bone, 4 from lung, and 1 from brain). Clinico-pathological data were collected from case records, the details of which are summarized in Table 1. Metastasis-free survival (MFS) was the primary outcome parameter analyzed. MFS was defined as the length of time from the start of treatment for cancer during which a patient was still alive and the cancer had not spread to other parts of the body. The Institutional Review Board of the hospital provided approval for the collection of archival samples. For this study, since only retrospective patient data and archived paraffin tissue blocks were used, a waiver of consent was obtained from the Research Advisory Council (RAC) under project RAC# 2110 031.

### 4.2. Tissue Microarray (TMA) Construction and Immunohistochemistry (IHC)

The tissue microarray (TMA) format was utilized for immunohistochemical analysis of the PTC samples. A TMA was constructed as previously described [40]. Briefly, a modified semiautomatic robotic precision instrument (Beecher Instruments, Woodland, WI, USA) was used to punch tissue cylinders with a diameter of 0.6 mm from representative tumor areas of donor tissue blocks and brought into recipient paraffin blocks. Two 0.6 mm cores of PTC were arrayed for each case.

Tissue microarray slides were processed and stained manually as described previously [41]. Primary antibodies against ZNF677 (HPA-024796, 1:200 dilution, pH 9.0, Sigma Aldrich, St. Louis, MI, USA) and p-AKT (736E11, 1:10 dilution, pH 9.0, Cell Signaling Technology, Danvers, MA, USA) were used. For ZNF677, a predominantly cytoplasmic staining was observed. The proportion of positively stained cells was calculated as a percentage for each core, and the scores were averaged across two tissue cores from the same tumor to yield a single percent staining score representing each cancer patient. For the purpose of statistical analysis, the scores were dichotomized. Cases showing any positive expression were classified as ZNF677-positive, and those with no expression were classified as ZNF677 loss. Scoring and cut-off for p-AKT were used as previously described by us [42]. Briefly, p-AKT was scored on an intensity scale ranging from 0 to 3 (0—no staining; 1—mild intensity; 2—moderate intensity; 3—strong intensity). All cases staining at the intensity levels 0 and 1 were grouped as p-AKT-negative, and all cases staining at the intensity levels 2 and 3 were grouped as p-AKT-positive. IHC scoring was done by two pathologists, blinded to the clinico-pathological characteristics. Discordant scores were reviewed together to achieve agreement.

### 4.3. Bisulfite Modification and Methylation-Specific PCR

Genomic DNA extracted from thyroid cancer cell lines was subjected to bisulfite modification using an EZ DNA Methylation kit (Zymo Research, Orange, CA, USA) as reported previously [43]. Methylation-specific PCR (MSP) was performed on bisulfite-treated DNA using primers specific for CpG islands in the ZNF677 promoter: forward primer, 5′-GAGGAGAGGTTCGGTAGTTC-3′ and reverse primer, 5′-TACGCGAATACACTAAAACGA-3′. For unmethylated DNA: forward primer, 5′-GTTTTTGTTGATTTGGAAGTGG-3′ and reverse primer, 5′-AACTAAAAACATCTTAAAACCACACC-3′. MSP products of ZNF677methylation and unmethylation were analyzed on 2% agarose gels and visualized under UV illumination after staining with ethidium bromide.

### 4.4. Cell Culture

Thyroid cancer cell lines 8505C, ML-1, TT2609, CGTH-W-1, CAL-62, BHT-101, and BCPAP cell lines were obtained from Deutsche Sammlung von Mikroorganismen und Zellkulturen (DSMZ, Braunschweig, Germany) , and TPC-1 was kindly provided by Dr. Bryan McIver (Department of Endocrinology, Mayo Clinic, Rochester, MN, USA). K1 cell line was purchased from the American Type Culture Collection (ATCC, Manassas, VA, USA). Cell were cultured in RPMI 1640 media supplemented with 10% fetal bovine serum (FBS), 100 Units/mL penicillin/streptomycin, and 100 Units/mL glutamine. These cell lines were authenticated in house using short-tandem-repeats PCR, and the results were in concordance with published data [44,45]. All experiments were performed using 5% FBS in RPMI 1640 medium.

### 4.5. Reagents and Antibodies

Antibodies against ZNF677 (NBP1-82677) were purchased from Novus Biologicals (Minneapolis, MN, USA). Antibodies against AKT (9272), Bcl2 (2872), Bcl-Xl (2762), E-cadherin (3195), Zeb1 (3396), MMP-2 (13132), MMP-9 (2270), PARP (9542), and cleaved caspase-3 (9664) as well as AKT siRNAs (6211 and 6510) were purchased from Cell Signaling Technologies (Danvers, MA, USA). N-cadherin (ab98952) and Twist (ab175430) antibodies were purchased from Abcam Inc. (Cambridge, MA, USA). Snail1 antibody (MA5-14801) and annexin V were purchased from Thermo Fisher Scientific (Rockford, IL, USA). Caspase-8 (66231A) antibody was purchased from BD PharMingen (San Diego, CA, USA). Phospho-AKT (sc-7985), caspase-3 (sc-56053), and GAPDH (sc-47724) antibodies were purchased from Santa Cruz Biotechnology, Inc. (Santa Cruz, CA, USA). The AKT inhibitor (MK2206) was purchase from Cayman (Ann Arbor, MI, USA).

### 4.6. Clonogenic Assay

The PTC cell lines were seeded at a density of 500 cells per well in a 6-well plate. After attachment, fresh growth medium was added, and the cells were allowed to grow for 8–10 days. Cell colonies were fixed with formaldehyde (4%) and stained with crystal violet (2% in 10% methanol). The number of colonies in each well were counted and photographed.

### 4.7. Annexin V Staining

PTC cells were treated with the indicated doses of TRAIL (Alexis Biochemicals, San Diego, CA, USA) or MK2206 (Selleckchem, Munich, Germany) for 48 h, and then the cells were harvested. The percentage apoptosis was measured by flow cytometry after staining with fluorescein-conjugated annexin-V and propidium iodide (PI), as described earlier [46].

### 4.8. Gene Silencing Using siRNA

Cells were transfected with AKT siRNAs and scrambled control siRNA using Lipofectamine 2000 (Invitrogen, Carlsbad, CA, USA) for 24 h, following which the lipid and siRNA complex was removed, and fresh growth medium was added. After 48 h of transfection, cells were used for immunoblotting.

### 4.9. Plasmid and Transfection

Plasmid DNA encoding human ZNF677 (RC207997) was purchased from Origene (Rockville, MD, USA). The overexpression of ZNF677 in PTC cells was performed using Lipofectamine™2000 (Invitrogen, Carlsbad, CA) according to the manufacturer’s protocol. Briefly, PTC cells were seeded in 6-well culture plates; when approximately 50% confluent, the cells were transfected with 4 µg of plasmid, and stably transfected clones were selected by G418 (Invitrogen, CA, USA); transfection efficacy was confirmed by immunoblotting.

### 4.10. Cell Invasion and Migration Assays

Cell invasion and migration assays were performed as described previously [45]. Briefly, cells after treatment were seeded into Trans-well inserts either uncoated (for migration assay) or coated (for invasion assay) with growth factor-reduced Matrigel for 24 h. After incubation, cells were stained with Diff-Quick stain set (Fisher Scientific, Pittsburg, PA, USA) and photographed under a fluorescent microscope.

### 4.11. Statistical Analysis

Contingency table analysis and Chi square tests were used to study the relationship between clinico-pathological variables and protein expression. The Kaplan–Meier method was used to generate metastasis-free survival curves, and the Mantel-Cox log-rank test was used to evaluate significance. Cox proportional hazards regression model was used to perform multivariate analysis, after adjusting for clinico-pathological variables like age, gender, stage, grade, site of tumor, and MSI status. The limit of significance for all analyses was defined as *p* < 0.05; two-sided tests were used in these calculations. The JMP14.0 (SAS Institute, Inc., Cary, NC, USA) software package was used for data analyses.

For all functional studies, data presented are means ± SD of triplicates in an independent experiment, which was repeated for at least two times with the same results. Student *t* test (two-tailed) was performed for statistical significance, with *p* < 0.05 used as the cut-off.

## 5. Conclusions

In conclusion, we report frequent ZNF677 expression loss in Middle Eastern PTC and its independent association with distant metastasis. Moreover, ZNF677 loss could serve as a molecular biomarker for predicting distant metastasis in PTC patients. Therefore, this work displays the promising prognostic and therapeutic potential of ZNF677 for aggressive PTCs.

## Figures and Tables

**Table 1:** Clinico-pathological variables of the patient cohort (*n* = 1235).

Clinico-Pathological Characteristics	n (%)
Age	
Median	37.5
Range (IQR)	28.7–50.5
<55 years	1005 (81.4)
≥55 years	230 (18.6)
Gender	
Female	938 (76.0)
Male	297 (24.0)
Histopathology	
Classical Variant	850 (68.8)
Follicular Variant	194 (15.7)
Tall Cell Variant	106 (8.6)
Others	85 (6.9)
Extra Thyroidal Extension	
Absent	640 (51.8)
Present	595 (48.2)
pT	
T1	318 (25.7)
T2	250 (20.2)
T3	517 (41.9)
T4	105 (8.5)
Unknown	45 (3.6)
pN	
N0	467 (37.8)
N1	633 (51.3)
Nx	135 (10.9)
pM	
M0	1091 (88.4)
M1	72 (5.8)
Mx	72 (5.8)
Stage	
I	1001 (81.1)
II	135 (10.9)
III	20 (1.6)
IVA	17 (1.4)
IVB	27 (2.2)
Unknown	35 (2.8)

IQR—Inter quartile range.

**Table 2:** Association of clinico-pathological characteristics with ZNF677 expression in PTC.

Clinico-Pathological Characteristics	Total		ZNF677 Loss		ZNF677 Normal		*p* Value
	No.	%	No.	%	No.	%	
No. of patients	1235		168	13.6	1067	86.4	
Age (years)							
<55	1005	81.4	136	13.5	869	86.5	0.8795
≥55	230	18.6	32	13.9	198	86.1	
Sex							
Female	938	75.9	122	13.0	816	87.0	0.2829
Male	297	24.1	46	15.5	251	84.5	
Extrathyroidal extension							
Absent	640	51.8	67	10.5	573	89.5	0.0008 *
Present	595	48.2	101	17.0	494	83.0	
pT							
pT1	318	26.7	34	10.7	284	89.3	0.2812
pT2	250	21.0	40	16.0	210	84.0	
pT3	517	43.5	70	13.5	447	86.5	
pT4	105	8.8	16	15.2	89	84.8	
pN							
pN0	467	42.5	67	14.4	400	85.6	0.5560
pN1	633	57.5	83	13.1	550	86.9	
pM							
pM0	1091	93.8	142	13.0	949	87.0	<0.0001 *
pM1	72	6.2	24	33.3	48	66.7	
Stage							
I	1001	83.4	134	13.4	867	86.6	0.0431 *
II	135	11.2	20	14.8	115	85.2	
III	20	1.7	3	15.0	17	85.0	
IVA	17	1.4	0	0.0	17	100.0	
IVB	27	2.3	8	29.6	19	70.4	
Histology Type							
Classical Variant	850	68.8	113	13.3	737	86.7	<0.0001 *
Follicular Variant	194	15.7	43	22.2	151	77.8	
Tall-Cell Variant	106	8.6	5	4.7	101	95.3	
Other variants	85	6.9	7	8.2	78	81.8	
phospho-AKT IHC							
High	499	40.4	116	23.3	383	76.7	<0.0001 *
Low	736	59.6	52	7.1	684	92.9	
Metastasis Free Survival							
5 years				89.2		95.1	0.0090 *

*—significant *p* value.

**Table 3:** Multivariate logistic regression analysis to assess the relationship between distant metastasis and clinico-pathological characteristics.

Clinico-Pathological Variables	Odds Ratio	95% CI	*p*-Value
Age ≥55 years (vs. <55 years)	0.66	0.25–1.72	0.3969
Sex Male (vs. Female)	2.31	1.17–4.56	0.0158 *
Extrathyroidal extension Present (vs. Absent)	2.07	0.90–4.75	0.0876
Histologic subtype Tall cell variant (vs. Other variants)	0.73	0.23–2.28	0.5868
Stage III–IV (vs. I–II)	33.62	12.37–91.39	<0.0001 *
ZNF677 IHC Loss (vs. positive)	2.60	1.20–5.62	0.0155 *

*—significant *p* value.

## Data Availability

The data presented in this study are available on request from the corresponding author.

## Supplementary Materials

The following are available online at https://www.mdpi.com/article/10.3390/ijms22157833/s1.
