# Supplementary material for: Loss of ZNF677 Expression Is an Independent Predictor for Distant Metastasis in Middle Eastern Papillary Thyroid Carcinoma Patients
Source: Int J Mol Sci. 2021 Jul 22;22(15):7833. doi: 10.3390/ijms22157833 (PMC8346014; doi:10.3390/ijms22157833)
Supplement: Supplementary file 1 [file ijms-22-07833-s001.zip › ijms-1230316-supplementary.pdf]

Supplementary Figure S1

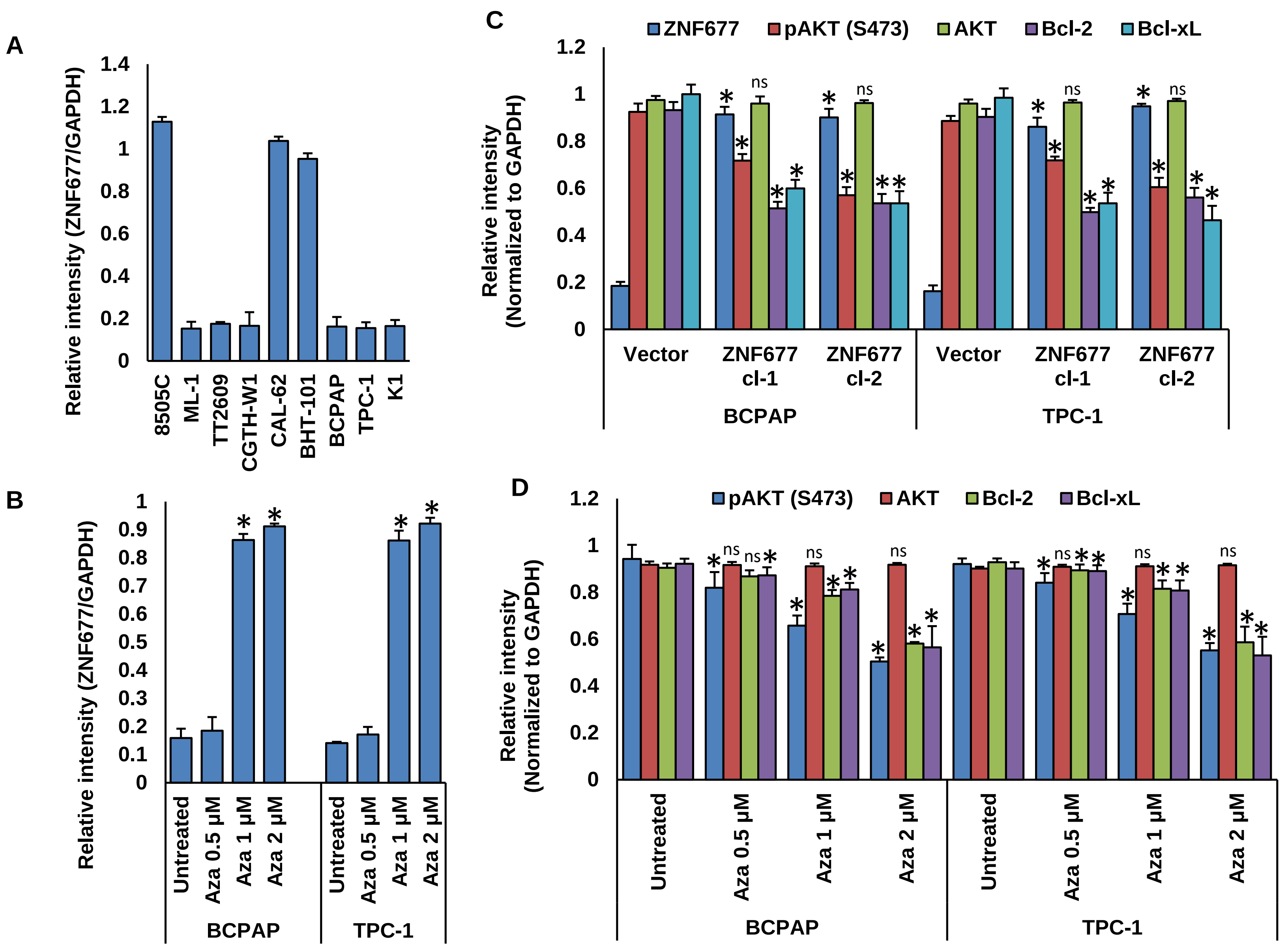

Supplementary Figure S2

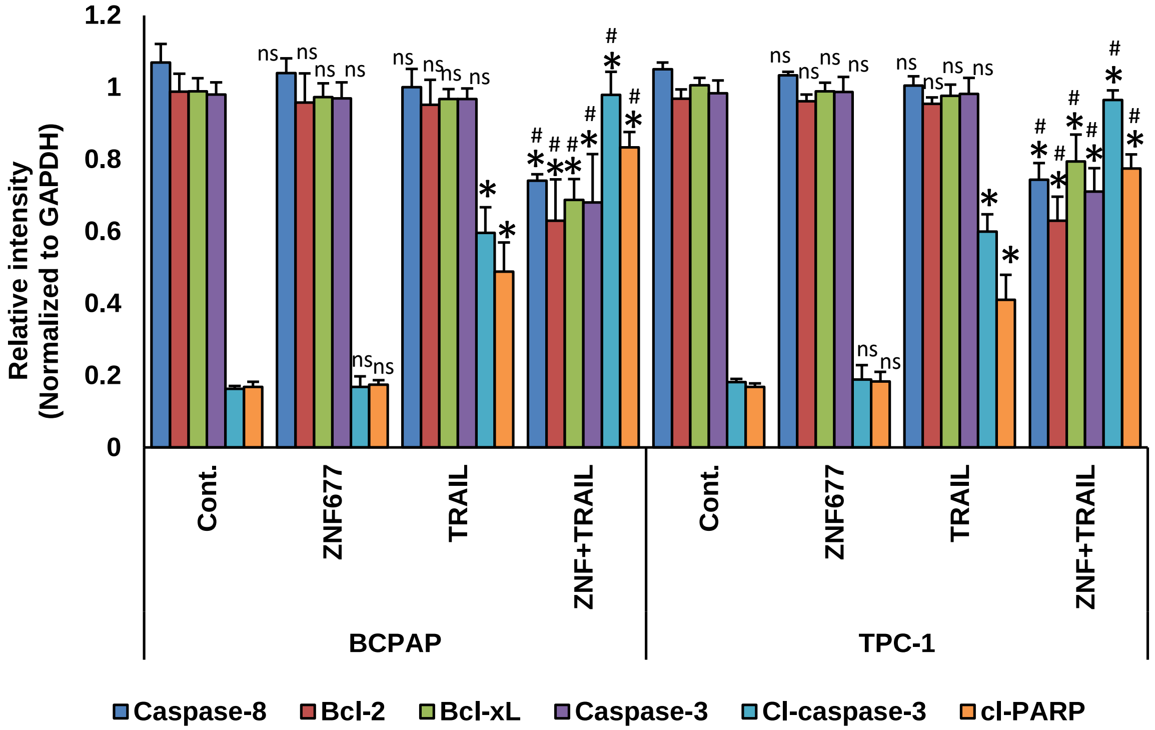

Supplementary Figure S3

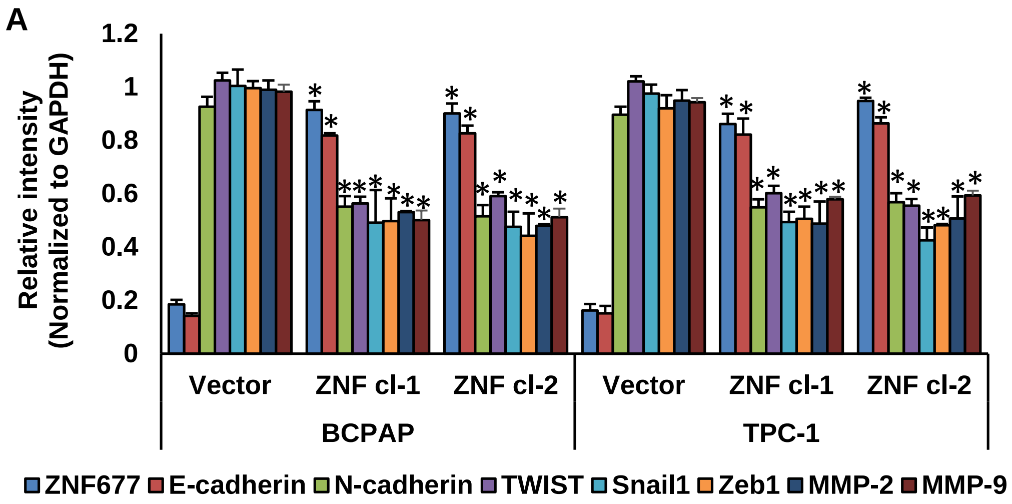

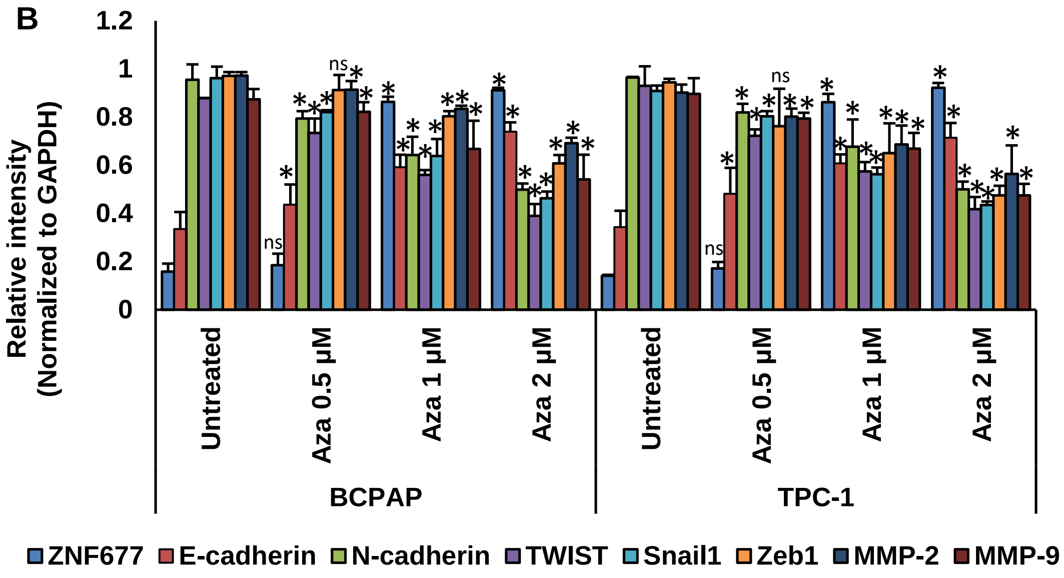

Supplementary Figure S4

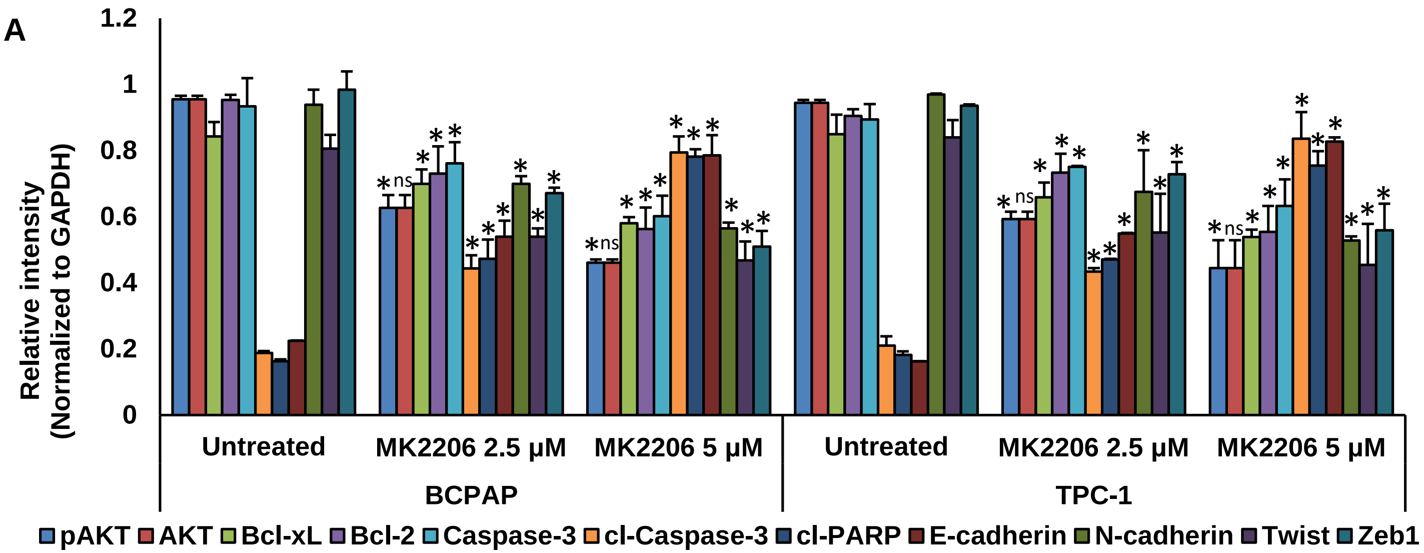

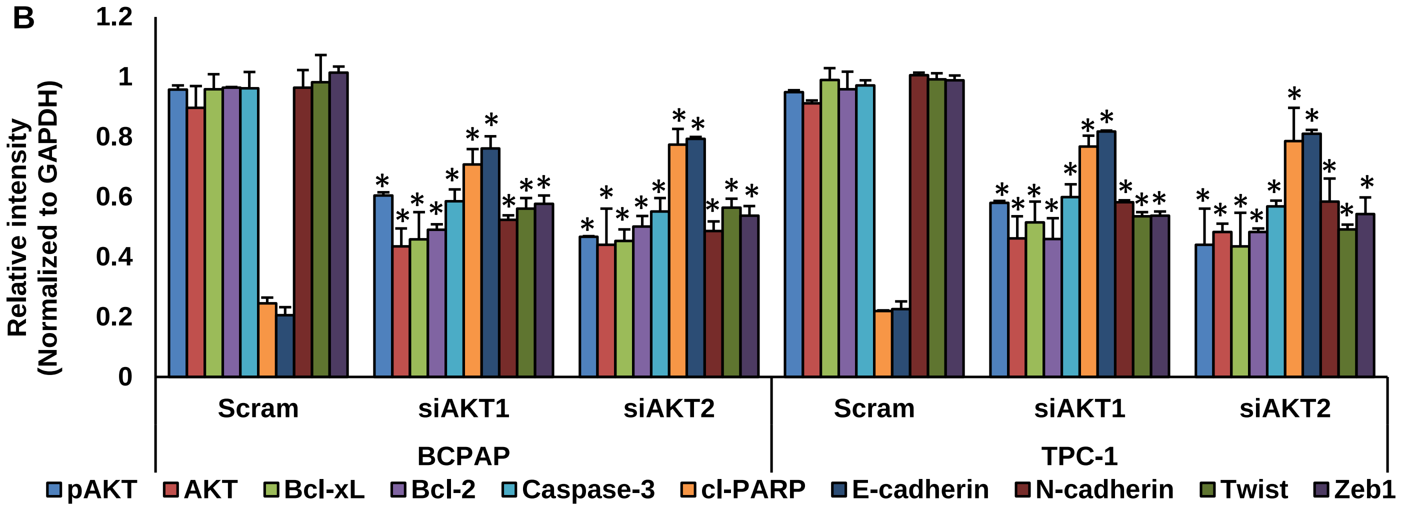

Supplementary Figure Legends: **Western blot quantification**

Supplementary Figure S1. (A) Basal expression of ZNF677 in a panel of thyroid cancer cell lines. Proteins were isolated from nine thyroid cancer cell lines and immunoblotted with antibodies against ZNF677 and GAPDH (n=3). **(B)** Demethylation of ZNF677 gene restored ZNF677 expression in BCPAP and TPC-1 cells. PTC cells were treated with different doses (0.5, 1 and 2 µM) of 5-aza-2'deoxycytidine for 72 hours and cells were lysed. Equal amounts of proteins were immunoblotted with antibodies against ZNF677 and GAPDH (n=3). **(C)** Ectopic expression of ZNF677 downregulate AKT phosphorylation and anti-apoptotic protein expressions. BCPAP and TPC-1 cell lines were transfected with either empty vector or ZNF677 cDNA and overexpression clones selected were immuno-blotted with antibodies against ZNF677, pAKT, AKT, Bcl-2, Bcl-xL and GAPDH as indicated (n=3). **(D)** Demethylation of ZNF677 gene downregulate AKT phosphorylation and anti-apoptotic protein expressions. PTC cells were treated with indicated doses of 5-aza-2'deoxycytidine for 72 hours and cells were lysed. Equal amounts of proteins were immunoblotted with antibodies against pAKT, AKT, Bcl2, Bcl-xL and GAPDH (n=3). Data presented in the bar graphs are the mean ± SD of three independent experiments. *Indicates a statistically significant difference compared to control with $p < 0.05$, ns= not significant.

Supplementary Figure S2. Forced expression of ZNF677 potentiates TRAIL-induced apoptosis in PTC cells. Cells were lysed and equal amounts of proteins were immunoblotted with antibodies against caspase-8, Bcl-2, Bcl-xL, caspase-3, cleaved- caspase-3, PARP and GAPDH. Data presented in the bar graphs are the mean ± SD of three independent experiments (n=3). *indicate statistically significant compared to empty vector control, #indicate statistically significant compared to TRAIL alone, with $p<0.05$, ns= not significant.

Supplementary Figure S3. (A-B) Forced expression of ZNF677 or Demethylation of ZNF677 gene attenuate EMT in PTC cells. ZNF677 overexpression clones or cells after treatment with indicated dose of 5-aza-2'deoxycytidine for 72 hours were immuno-blotted with antibodies against ZNF677, E-cadherin, N-cadherin, Twist, Snail1, Zeb1, MMP-2, MMP-9 and GAPDH. Data presented in the bar graphs are the mean ± SD of three independent experiments (n=3). *Indicates a statistically significant difference compared to control with $p < 0.05$, ns= not significant.

Supplementary Figure S4. (A-B) Inhibition of AKT reduce the markers of cell growth and EMT in PTC cells. PTC cells were treated with indicated doses of MK2206 or transfected two different AKT siRNA's (100 nM) for 48 hours. Cells were lysed and equal amounts of proteins were immunoblotted with antibodies against pAKT, AKT, Bcl-2, Bcl-xL, caspase-3, cleaved- caspase-3, PARP, E-cadherin, N-cadherin, Twist, Zeb1 and GAPDH (n=3). *Indicates a statistically significant difference compared to control with $p < 0.05$, ns= not significant.
